# Potentiometric Solid-Contact K^+^ Ion-Selective Electrodes Based on the KMnFe(CN)_6_ Transducer

**DOI:** 10.3390/membranes16050156

**Published:** 2026-04-29

**Authors:** Huali Deng, Zhanhao Liu, Li Niu, Shiyu Gan

**Affiliations:** 1 Center for Advanced Analytical Science, Guangzhou Key Laboratory of Sensing Materials & Devices, Guangdong Engineering Technology Research Center for Photoelectric Sensing Materials & Devices, School of Chemistry and Chemical Engineering, Guangzhou University, Guangzhou 510006, China; 2 School of Chemical Engineering and Technology, Sun Yat-sen University, Zhuhai 519082, China

**Keywords:** ion-selective electrode, solid-contact, potassium ion detection, transducer

## Abstract

Solid-contact ion-selective electrodes (SC-ISEs) are typically constructed using ion-selective membrane (ISM)-based configurations. However, such structures often suffer from water-layer formation and the weak mechanical stability of the ISM. Herein, we report an ISM-free K^+^-SC-ISE based on a Prussian blue analogue transducer, KMnFe(CN)_6_, eliminating the need for a conventional ionophore-based ISM layer. KMnFe(CN)_6_ was synthesized via a one-step citrate-assisted co-precipitation method. The material functions as a bifunctional transducer, in which the open framework structure with ion-transport channels enables selective K^+^ recognition, while the redox-active Mn centers facilitate ion-to-electron transduction. The fabricated KMnFe(CN)_6_-based K^+^ sensor exhibits a near-Nernstian response with a sensitivity of 52.3 ± 1.0 mV dec^−1^ and a rapid response time of 25 s. The linear range and limit of detection were determined to 10^−4^ to 10^−1^ M and 5.8 × 10^−5^ M, respectively. The sensor also demonstrates selectivity to representative interfering ions, with log *K_ij_* of −2.39 ± 0.12 (Na^+^), −2.86 ± 0.09 (Li^+^), −3.06 ± 0.09 (Ca^2+^), −2.74 ± 0.12 (Mg^2+^) and −0.95 ± 0.08 (NH_4_^+^). By eliminating the ISM layer, the water-layer effect is effectively avoided, resulting in excellent long-term stability with a potential drift of 57.2 ± 6.1 μV h^−1^ over 7 days. The sensor was further applied to the analysis of K^+^ in real lake water samples, where the measured concentration showed good agreement with ion chromatography results. This work provides an ISM-free SC-ISE strategy for ion analysis in water environments.

## 1. Introduction

Solid-contact ion-selective electrodes (SC-ISEs) are a class of miniaturized electrochemical sensors widely used for the quantitative analysis of ionic species in environments [1,2,3,4,5], biomedicals [6,7,8,9,10] and agriculture [11,12,13,14,15]. These sensors feature rapid response, portability, and the capability for on-site monitoring. Conventional SC-ISEs typically adopt a sandwich structure composed of a conductive substrate, a solid-contact (SC) layer, and an ion-selective membrane (ISM) [16,17,18]. In this configuration, the SC layer converts ionic concentration changes into measurable electronic potential signals, while the ISM selectively recognizes and binds the target ions. However, the interface between the SC layer and the ISM is susceptible to the formation of a nanoscale water layer [19,20,21], which can lead to significant potential drift and reduced measurement accuracy. To address this issue, various advanced transducer materials have been explored as SC layers from classic polymers to diverse nanomaterials [17,22,23]. Although these developments have significantly improved SC-ISE performance, the presence of nanoscale water layers and the weak mechanical strength of the ISM remain major challenges in this field.

State-of-the-art potentiometric K^+^ sensors are also predominantly based on ISM-based SC-ISEs with various solid-contact transducers developed [24]. In these sensors, K^+^ recognition is achieved through ionophores incorporated in the ISM, such as valinomycin, while the transduction of ionic signals into electronic signals is accomplished by the SC transducer. Early conducting polymers, such as PEDOT(PSS), have been widely used due to their facile preparation and high electronic and ionic conductivity [25,26,27]. However, their intrinsic hydrophilicity can promote the formation of water layers at the interface. To mitigate this problem, hydrophobic conducting polymers have been developed by grafting lipophilic functional groups [28,29,30,31]. Various nanomaterials, including carbon materials, metal nanoparticles and metal oxide materials, have been explored as transducer materials owing to their hydrophobic properties [32,33,34]. In addition, their large surface area can increase the interfacial capacitance, thereby enhancing potential stability. However, our recent work has indicated that interfacial hydrophobicity is negatively correlated with interfacial capacitance, suggesting that both factors must be carefully balanced [35]. Recently, emerging nanomaterials, such as metal–organic frameworks (MOFs) [36,37,38,39] and hybrid nanocomposites [40,41,42,43,44], have further improved sensor stability. Nevertheless, most existing K^+^-SC-ISEs still rely on the conventional ISM structure. The organic ISMs continue to suffer from limited mechanical strength and potential component leakage [45].

In this work, we report an ISM-free K^+^-SC-ISEs based on a bifunctional Prussian blue analogue (PBA) transducer, KMnFe(CN)_6_, without the use of a conventional ISM layer. PBAs are mixed electronic–ionic conductors whose three-dimensional framework structures enable reversible ion intercalation and deintercalation [46]. Furthermore, the reversible redox reactions of Mn and Fe facilitate efficient ion-to-electron signal transduction. The fabricated KMnFe(CN)_6_-based ISM-free SC-ISE exhibits a near-Nernstian response toward K^+^. Its selectivity is comparable to that of crown-ether-based ionophores and approaches that of valinomycin-based membranes. The sensor also demonstrates a rapid response time and excellent potential stability. Finally, the sensor enables accurate determination of K^+^ in real lake water samples. This work highlights that the conventional three-layer SC-ISE architecture can be simplified to a two-layer structure through the development of ISM-free SC-ISEs.

## 2. Materials and Methods

### 2.1. Materials Preparation

KMnFe(CN)_6_ was synthesized via a chemical co-precipitation method using potassium citrate as a chelating agent, following a previously reported procedure with slight modification. Briefly, 10 mM MnSO_4_·H_2_O and 0.15 M potassium citrate were dissolved in 50 mL of deionized water to prepare solution A. Separately, 10 mM K_4_Fe(CN)_6_·3H_2_O was dissolved in deionized water to form solution B. Solution A was then slowly added dropwise into solution B under continuous magnetic stirring. The reaction mixture was stirred for 12 h and subsequently aged for another 12 h at room temperature. The resulting white precipitate was collected by centrifugation, washed several times with deionized water and ethanol, and finally dried in a vacuum oven at 80 °C for 12 h to obtain KMnFe(CN)_6_ powder.

### 2.2. Materials Characterization

The crystal structure of KMnFe(CN)_6_ was characterized by powder X-ray diffraction (XRD, Rigaku Ultima IV, Tokyo, Japan) with a scanning range of 10–80°. The elemental composition and content were determined using inductively coupled plasma mass spectrometry (ICP–MS, Shimadzu 2030 LF, Kyoto, Japan). The water of crystallization was analyzed by thermogravimetric analysis (TGA, TA Instruments TGA55, New Castle, DE, USA), performed from room temperature to 500 °C at a heating rate of 10 °C min^−1^ under a nitrogen atmosphere. The chemical bonding characteristics were identified using Fourier transform infrared spectroscopy (FTIR, Thermo Fisher Scientific, Nicolet iN10, Waltham, MA, USA). The valence states of the elements were investigated by X-ray photoelectron spectroscopy (XPS, Thermo Scientific K-Alpha, East Grinstead, UK). The morphology and microstructure were examined using transmission electron microscopy (TEM, FEI Talos F200x, Eindhoven, The Netherlands) equipped with energy-dispersive spectroscopy (EDS). The lake water was collected near the Guangzhou University and was simply filtered before use. The ionic composition and concentrations of real lake water samples were analyzed using an ion chromatography (IC) system (Qingdao Shine, CIC-D120, Qingdao, China).

### 2.3. Electrode Preparation

The KMnFe(CN)_6_-based electrodes were prepared as follows. Glassy carbon electrodes (GCEs, diameter = 5 mm) were used as the conductive substrates. Prior to modification, the GCEs were sequentially polished with 0.5 μm and 0.3 μm alumina powders and then ultrasonically cleaned in deionized water and ethanol. A dispersion of KMnFe(CN)_6_ was prepared by dispersing 20 mg of KMnFe(CN)_6_ powder and 4 mg of poly(vinylidene fluoride) (PVDF) in 1 mL of N-methyl-2-pyrrolidone (NMP). The KMnFe(CN)_6_-based K^+^ sensors were fabricated by drop-casting 10 μL of the dispersion onto the surface of the GCEs, followed by drying at 60 °C for 3 h.

### 2.4. Potentiometric Measurements

All potentiometric measurements were conducted using a two-electrode system with an EMF6 multichannel potentiometer. The KMnFe(CN)_6_/GCE served as the working electrode, while a saturated calomel electrode (SCE) equipped with an external salt bridge (1 M LiOAc) was used as the reference electrode. The electrochemical impedance spectrum was measured by a Gamry workstation (reference 600+, USA) using a three-electrode system. The Pt wire was used as the counter electrode. Prior to measurements, the KMnFe(CN)_6_/GCE was activated in 10^−4^ M KCl solution for 2 h to ensure a stable potentiometric response. Calibration curves for the K^+^-selective electrode were obtained by measuring the electromotive force (EMF) in KCl solutions with concentrations ranging from 10^−7^ to 10^−1^ M. The ion activities were calculated according to the extended Debye–Hückel equation. The potentiometric selectivity coefficients (*K_ij_*) toward interfering ions were determined using the separate solution method (SSM) and the *K_ij_* was calculated according to the Nikolsky-Eisenman equation [47]

(1) $${log\;K}_{ij}=\frac{E_{j}^{o}-E_{i}^{o}}{2.303\;RT}z_{i}F$$

$E_{j}^{o}$ and $E_{i}^{o}$ represent the standard electrode potentials of interfering and primary ions, respectively. *z_i_* means the charge of primary K^+^. *R*, *T* and *F* have their own meanings. The long-term stability of the electrode was evaluated by continuously monitoring the potential in a 10^−1^ M KCl solution for 7 days.

## 3. Results and Discussion

### 3.1. ISM-Free K^+^-SC-ISEs Based on KMnFe(CN)_6_

**Figure 1:**
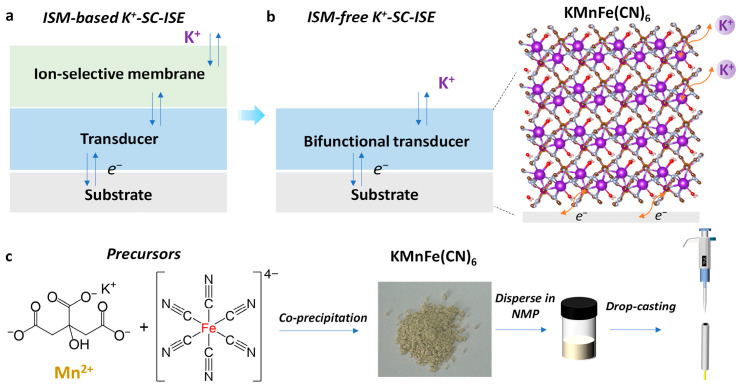
Ion-selective-membrane-free SC-ISE for K^+^ detection: (**a**) Schematic illustration of conventional membrane-based SC-ISEs for K^+^ detection, consisting of three layers: an ion-selective membrane (ISM), a solid-contact (SC) transducer, and a conductive substrate. (**b**) Schematic illustration of the proposed ISM-free K^+^-SC-ISE based on KMnFe(CN)_6_ as a bifunctional transducer, eliminating the need for an ionophore-based ISM layer. (**c**) Schematic illustration of the synthesis of the KMnFe(CN)_6_ transducer and the fabrication process of KMnFe(CN)_6_-based SC-ISEs for K^+^ detection.

As mentioned above, conventional ISM-based K^+^-SC-ISEs typically adopt a three-layer sandwich structure consisting of a conductive substrate, a solid-contact transducer layer, and an ion-selective membrane (ISM), in which each layer performs a specific function (Figure 1a). However, this architecture suffers from several inherent limitations, including the formation of an interfacial water layer, the high cost of ionophores, and the poor mechanical stability of the organic ISM layer. In this work, we propose an ISM-free K^+^-SC-ISEs that employs KMnFe(CN)_6_ as a bifunctional layer capable of simultaneously achieving K^+^-specific recognition and ion-to-electron transduction (Figure 1b).

The selectivity toward K^+^ arises from the reversible transfer of K^+^ ions between the crystal lattice of KMnFe(CN)_6_ and the external solution, establishing a K^+^ equilibrium at the interface. Meanwhile, ion-to-electron signal transduction is mediated by the redox-active Mn centers within the framework. Overall, the sensing process can be described as a K^+^ ion-coupled electron transfer mechanism. By eliminating the ISM layer, the conventional three-layer SC-ISE architecture is simplified into a two-layer structure. This simplified configuration not only reduces structural complexity but also improves mechanical robustness and may enhance the applicability of the sensor for ion analysis in complex environmental samples.

### 3.2. Structure and Compositions of Transducer

**Figure 2:**
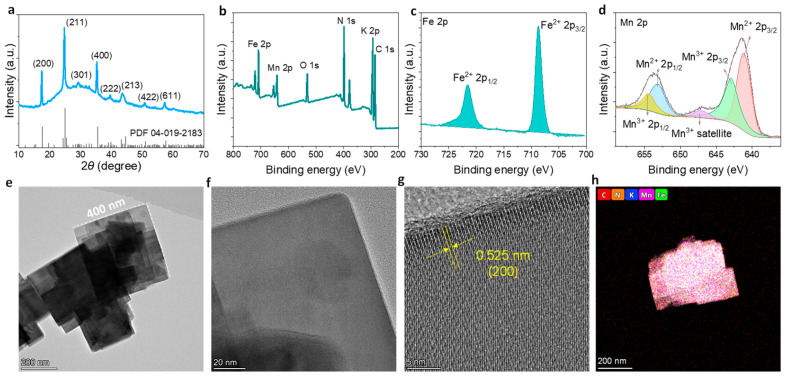
Structural and compositional characterization of KMnFe(CN)_6_ transducers: (**a**) XRD pattern of KMnFe(CN)_6_ compared with the standard PDF card of K_2_MnFe(CN)_6_·2H_2_O (PDF#04-019-2183). (**b**–**d**) XPS characterization of KMnFe(CN)_6_, including (**b**) survey spectrum, (**c**) high-resolution Fe 2p spectrum, and (**d**) high-resolution Mn 2p spectrum. (**e**–**g**) TEM and high-resolution TEM (HRTEM) images of KMnFe(CN)_6_. (**h**) Corresponding elemental mapping images showing the distribution of K, Mn, Fe, C, and N elements.

The KMnFe(CN)_6_ transducer material was synthesized via a chemical precipitation method using Mn^2+^ and K_4_Fe(CN)_6_ as precursors (Figure 1c). Potassium citrate was introduced as a coordinating ligand to regulate the release of Mn^2+^, thereby slowing the precipitation process and facilitating the formation of uniform KMnFe(CN)_6_ particles. The crystal structure of the synthesized KMnFe(CN)_6_ was first characterized by X-ray diffraction (XRD) (Figure 2a). Well-defined diffraction peaks were observed and matched well with the standard PDF card (#04-019-2183) [48] for K_2_MnFe(CN)_6_·2H_2_O. These results indicate that the prepared material possesses an orthorhombic crystal structure (space group Pmn2_1_), in which the C atoms coordinate with Fe cations and the N atoms coordinate with Mn cations. The FT-IR spectrum of KMnFe(CN)_6_ is shown in Figure S1. Prominent absorption peaks at 2073 and 588 cm^−1^ are attributed to the stretching and bending vibration of the Mn^2+^–N≡C–Fe^2+^ bond, confirming the successful formation of the PBA framework. In addition, characteristic absorption peaks at 3446 cm^−1^ and 1631 cm^−1^ indicate the presence of crystal water in the material. The thermogravimetric analysis (TGA) curve shows a weight loss of 7.35% below 250 °C (Figure S2), corresponding to the loss of crystal water in the temperature range of 150–250 °C. The precise elemental composition of KMnFe(CN)_6_ was determined by inductively coupled plasma mass spectrometry (ICP–MS) (Table S1). The ICP–MS results reveal that the molar ratio of K:Mn:Fe in the material is 1.58:1:0.81. Based on the combined results from the ICP–MS and TGA analyses, the chemical composition is determined to be K_1.58_Mn[Fe(CN)_6_]_0.81_·1.27H_2_O (Table S1).

X-ray photoelectron spectroscopy (XPS) analysis was conducted to investigate the chemical valence states of Fe and Mn in KMnFe(CN)_6_. The survey XPS spectrum confirms the presence of K, Mn, Fe, C, N, and O elements, with characteristic peaks corresponding to K 2p, Mn 2p, Fe 2p, C 1s, N 1s, and O 1s (Figure 2b). The O signal originates from crystal water molecules within the structure. The high-resolution Fe 2p spectrum shows two characteristic peaks located at 708.7eV and 721.5 eV (Figure 2c), which are assigned to Fe^2+^ 2p_3/2_ and Fe^2+^ 2p_1/2_, respectively. In the high-resolution Mn 2p spectrum (Figure 2d), the peaks at 641.2/642.9 eV and 653.2/654.5 eV correspond to the Mn^2+/3+^ 2p_3/2_ and Mn^2+/3+^ 2p_1/2_ components, respectively, confirming the coexistence of Mn^2+^ and Mn^3+^ in the material. The presence of mixed-valence Mn species provides redox-active sites that facilitate ion-to-electron transduction, which is essential for the potentiometric response of the electrode.

The morphology and elemental distribution of KMnFe(CN)_6_ were further characterized by transmission electron microscopy (TEM) and elemental mapping (Figure 2e–h). The material exhibits a typical cubic morphology with an average lateral size of approximately 400 nm (Figure 2e). High-resolution TEM reveals a well-defined orthorhombic edge (Figure 2f) and clear lattice spacing of ~0.525 nm (Figure 2g), which corresponds to the (200) crystallographic plane. Elemental mapping images show that K, Mn, Fe, C, and N are uniformly distributed throughout the particles, further confirming the successful synthesis of a homogeneous KMnFe(CN)_6_ material (Figure 2h).

### 3.3. Potentiometric K^+^ Response Performances

**Figure 3:**
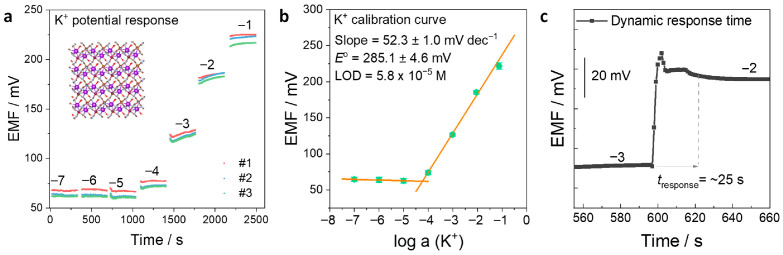
Potentiometric response of KMnFe(CN)_6_-based SC-ISEs toward K^+^: (**a**) Potential responses of KMnFe(CN)_6_ electrodes measured in KCl solutions with concentrations ranging from 10^−7^ to 10^−1^ M. The numbers on the response curves indicate the logarithmic concentrations of K^+^. Three independent electrodes were tested (*n* = 3). (**b**) Corresponding calibration curves between EMF and the calibrated activity of K^+^. (**c**) Dynamic potentiometric response curve used to evaluate the response time of the KMnFe(CN)_6_ electrode during a stepwise concentration change in K^+^ from 10^−3^ to 10^−2^ M.

The potentiometric response toward the primary ion K^+^ was first evaluated (Figure 3). The electromotive force (EMF) was measured in KCl solutions with concentrations ranging from 10^−7^ to 10^−1^ M (Figure 3a). The EMF exhibits a noticeable response starting from 10^−5^ M and increases with increasing K^+^ concentration. Linear fitting of the calibration curve yields a sensitivity of 52.3 ± 1.0 mV dec^−1^ and a standard electrode potential (*E*^o^) of 285.1 ± 4.6 mV (Figure 3b). The slope is close to the theoretical Nernstian response for a monovalent ion. In addition, the small standard deviation (SD) of *E*^o^ indicates good reproducibility of the electrode. The limit of detection (LOD) was calculated to be 5.8 × 10^−5^ M, which meets the requirements for many practical analytical applications, such as the determination of mM-level K^+^ in a water environment.

The response time of the electrode was further investigated using dynamic potential response measurements (Figure 3c), in which the EMF was continuously recorded. When the K^+^ concentration was changed from 10^−3^ M to 10^−2^ M, a transient potential spike was observed due to solution stirring upon the addition of the concentrated K^+^ solution. The response time was determined to be approximately 25 s. This response time is comparable to that of conventional ISM-based K^+^-SC-ISEs, indicating that the ISM-free KMnFe(CN)_6_ electrode maintains a rapid response capability. The fast response can be attributed to the open three-dimensional framework structure of KMnFe(CN)_6_, which enables rapid diffusion and intercalation of K^+^ ions at the electrode/solution interface, as well as the direct ion-to-electron transduction provided by the bifunctional layer without the mass-transfer resistance associated with an ISM.

### 3.4. Potentiometric Responses Toward Interfering Ions and Selectivity

**Figure 4:**
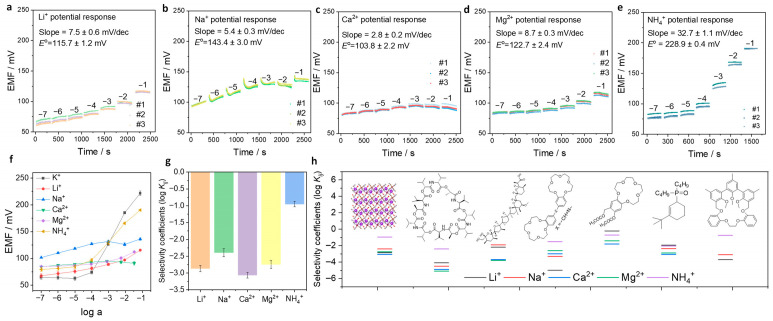
Potentiometric responses toward interfering ions and selectivity: (**a**–**e**) Potentiometric response curves of KMnFe(CN)_6_ electrodes toward representative interfering ions: Li^+^, Na^+^, Ca^2+^, Mg^2+^ and NH_4_^+^. The numbers on the curves indicate the logarithmic concentrations of the corresponding interfering ions. The slope and intercept (*E*^o^) were determined from Figures S3–S7. (**f**) Comparison of potentiometric response curves between the primary ion K^+^ and interfering ions. (**g**) Calculated potentiometric selectivity coefficients (log *K_ij_*) of KMnFe(CN)_6_ electrodes toward different interfering ions. (**h**) Comparison of selectivity coefficients between KMnFe(CN)_6_ electrodes and a few K^+^ ionophores. The selectivity coefficients data for the ionophores from left to right cited from references [49,50,51,52,53,54].

Selectivity is a key parameter for evaluating the performance of SC-ISEs. The previous results demonstrated a near-Nernstian response sensitivity toward the primary ion K^+^. In this section, the potentiometric responses toward several representative interfering ions were further investigated. Li^+^, Na^+^, Ca^2+^, and Mg^2+^ were selected as typical interfering ions. For instance, in the case of Li^+^, the response curves show that the electrode exhibits weak responses across the entire concentration range (Figure 4a), resulting in only a slight response sensitivity of 7.5 ± 0.6 mV dec^−1^. The corresponding standard electrode potential (*E*^o^) was determined to be 115.7 ± 1.2 mV, which is significantly lower than that observed for the primary ion K^+^. Similar behaviors were observed for Na^+^, Ca^2+^, and Mg^2+^ (Figure 4b–d). Their response sensitivities were determined to be 5.4 ± 0.3 mV dec^−1^ for Na^+^, 2.8 ± 0.2 mV dec^−1^ for Ca^2+^, and 8.7 ± 0.3 mV dec^−1^ for Mg^2+^. These values are substantially lower than the sensitivity toward K^+^, indicating limited interference from these ions. NH_4_^+^ has an ionic radius similar to that of K^+^ and is therefore expected to exhibit a certain level of response. As shown in Figure 4e, the response sensitivity toward NH_4_^+^ was determined to be 32.7 ± 1.1 mV dec^−1^. Although this value is higher than those observed for other interfering ions, it remains significantly lower than that for the primary ion, K^+^ (52.3 ± 1.0 mV dec^−1^, Figure 3b). In addition, the *E*^o^ for NH_4_^+^ (228.9 ± 0.4 mV) is lower than that for K^+^ (285.1 ± 4.6 mV), further indicating preferential selectivity toward K^+^.

The potentiometric response curves of the interfering ions are compared with that of the primary ion K^+^ in Figure 4f. The EMF values obtained for all interfering ions are lower than those for K^+^. Based on the differences in the *E*^o^, the potentiometric selectivity coefficients (log *K_ij_*) were quantitatively calculated using the Nikolsky–Eisenman equation. The KMnFe(CN)_6_ electrodes exhibit selectivity coefficients of log *K_ij_* = −2.39 ± 0.12 for Na^+^, −2.86 ± 0.09 for Li^+^, −3.06 ± 0.09 for Ca^2+^, −2.74 ± 0.12 for Mg^2+^ and −0.95 ± 0.08 for NH_4_^+^ (Figure 4f). For other potential interfering ions, such as H^+^, the response was evaluated separately. Since the [Fe(CN)_6_]^4−^ framework in KMnFe(CN)_6_ is unstable under acidic or alkaline conditions, and considering that most natural water environments are neutral or slightly alkaline, the pH effect was investigated within the range of 7–8 (Figure S8). The potential fluctuation was found to be less than 2 mV, which satisfies typical measurement requirements. In addition, interference from Mn^2+^ was evaluated due to its presence in KMnFe(CN)_6_ (Figure S9). The potentiometric response toward Mn^2+^ exhibited a sensitivity of 32.2 ± 0.6 mV dec^−1^, which is significantly lower than that for the primary ion, K^+^ (Figure 3b). The *E*^o^ for Mn^2+^ was determined to be 247.4 ± 1.4 mV, also lower than that for K^+^. The corresponding selectivity coefficient was calculated as log *K_ij_* = −0.64 ± 0.06. Although the selectivity toward Mn^2+^ is relatively limited, its concentration in natural water environments is also typically at the micromolar level. Therefore, Mn^2+^ is not expected to cause significant interference under practical conditions.

Finally, the analytical performance of the KMnFe(CN)_6_-based K^+^-SC-ISE was compared with that of conventional ISM-based K^+^-ISEs (Table S2). The ISM-free KMnFe(CN)_6_ electrode exhibits near-Nernstian sensitivity comparable to that of traditional ISM-based systems. The linear range (10^−4^ to 10^−1^ M) and LOD (5.8 × 10^−5^ M) are somewhat inferior to those of conventional ISM-based SC-ISEs; however, these parameters remain sufficient for K^+^ determination in most natural environments. In terms of selectivity, although the performance is lower than that of the widely used valinomycin ionophore [49], it is comparable to several other K^+^ ionophores (Figure 4g), including polyether ionophore antibiotics (e.g., nigericin) [50], crown ether-based ionophores [51,52], organophosphine ligands [53], and calixarene-based molecules [54,55]. For example, hemispherand ionophore 3 exhibits selectivity coefficients of log *K*(K^+^/Na^+^) = −3.1, log *K*(K^+^/Li^+^) = −3.7, and log *K*(K^+^/NH_4_^+^) = −0.75 [54]. The KMnFe(CN)_6_ electrode demonstrates approaching selectivity to this class of K^+^ ionophores.

The ion selectivity of state-of-the-art ISM-based SC-ISEs generally relies on ionophores that interact with target metal ions through coordination effects. In contrast, the ion recognition mechanism of KMnFe(CN)_6_ is governed by an ion intercalation equilibrium between the crystal lattice and the external solution, for example: K^+^ (lattice) ⇆ K^+^ (solution). To further investigate this mechanism, electrochemical impedance spectroscopy (EIS) measurements were conducted for KMnFe(CN)_6_ electrodes in various electrolyte solutions (Figure S10a). The imaginary component of the impedance at low frequency (0.01 Hz) reflects the capacitance of the SC-ISE. The low-frequency capacitance (*C_lf_*) was calculated using the following equation [56]:

(2) $$C_{lf}=-\frac{1}{2\pi fZ^{\prime \prime }}$$

where f is the frequency (0.01 Hz) and Z″ is the imaginary component of the impedance. Based on the Z″ values obtained from the EIS measurements in different electrolytes (Figure S10a), the *C_lf_* values were determined to 34.4 μF (NH_4_^+^), 29.5 μF (K^+^), 31.1 μF (Na^+^), 37.7 μF (Li^+^), 29.3 μF (Mg^2+^) and 38.1 μF (Ca^2+^). These values are relatively similar, indicating that the capacitance is primarily governed by the KMnFe(CN)_6_ transducer. The bulk membrane resistance and the charge-transfer resistance associated with ion intercalation were derived from the real part of the impedance at low frequency (Z′). The corresponding resistance values were 197.3 kΩ (NH_4_^+^), 203.6 kΩ (K^+^), 264.7 kΩ (Na^+^), 287.0 kΩ (Li^+^), 271.1 kΩ (Mg^2+^) and 277.7 kΩ (Ca^2+^). Notably, NH_4_^+^ and K^+^ exhibit similar resistance values, confirming that NH_4_^+^ is the most significant interfering ion for K^+^ detection. For the Li^+^, Na^+^, Ca^2+^ and Mg^2+^, their selectivity coefficients are basically consistent with the order of their charge transfer resistance (Figure S10b). These EIS results suggest that the ion selectivity of KMnFe(CN)_6_ electrodes is governed by ion intercalation resistance, which fundamentally differs from the coordination-based ion recognition mechanism of conventional ionophores. In addition to ion recognition, it intrinsically enables ion-to-electron transduction, functioning as a bifunctional transducer and thereby significantly simplifying the conventional three-layer electrode architecture.

### 3.5. Long-Term Stability

**Figure 5:**
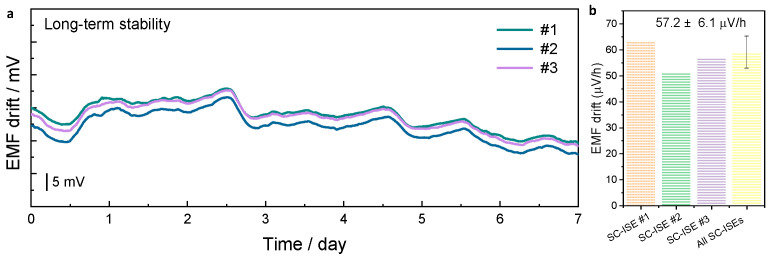
Long-term stability of SC-ISEs: (**a**) The EMF drifts for four individual KMnFe(CN)_6_-based SC-ISEs in 0.1 M KCl for a duration of 7 days. (**b**) Calculated EMF drifts for each electrode and average potential drift (*n* = 3).

After evaluating the sensitivity and selectivity of the KMnFe(CN)_6_-based SC-ISEs, the long-term stability of the electrodes was further investigated. First, the water-layer effect was examined, although no ISM layer is present in the proposed KMnFe(CN)_6_ electrodes. A standard three-stage testing protocol was employed (Figure S11). The electrodes were initially tested in 0.1 M KCl (stage I), where a stable potential was observed. Upon switching the solution to 0.1 M LiCl (stage II), a slight initial potential decrease was observed, likely due to the establishment of ion equilibrium, followed by a stable potential. When the electrodes were returned to 0.1 M KCl (stage III), the potential recovered to its original value observed in stage I. These results confirm the absence of a water-layer effect in the KMnFe(CN)_6_ electrodes. To further assess their long-term operational stability, continuous potentiometric measurements were performed over a period of 7 days (Figure 5a). According to the EMF drift curves, all SC-ISEs exhibit stable potentials throughout the testing period. The potential drift values were calculated from the difference between the initial and final EMF values. The three electrodes showed drift rates of 63.4, 51.3, and 57.0 μV h^−1^, respectively (Figure 5b). The average potential drift was determined to be 57.2 ± 6.1 μV h^−1^. This stability is comparable to, or even better than, that of representative ISM-based SC-ISEs employing hydrophobic transducers, such as polypyrrole doped with perfluorooctanesulfonate (PPy–PFOS, 69 μV h^−1^) [29]. Unlike traditional ionophore-containing polymeric ISM layers, which often suffer from mechanical fragility and component leakage, the ISM-free KMnFe(CN)_6_ electrode provides improved mechanical and operational robustness under complex and variable environmental conditions. In the following section, the performance of the sensor for K^+^ detection in real samples is further evaluated.

### 3.6. Practical Sample Analysis

**Figure 6:**
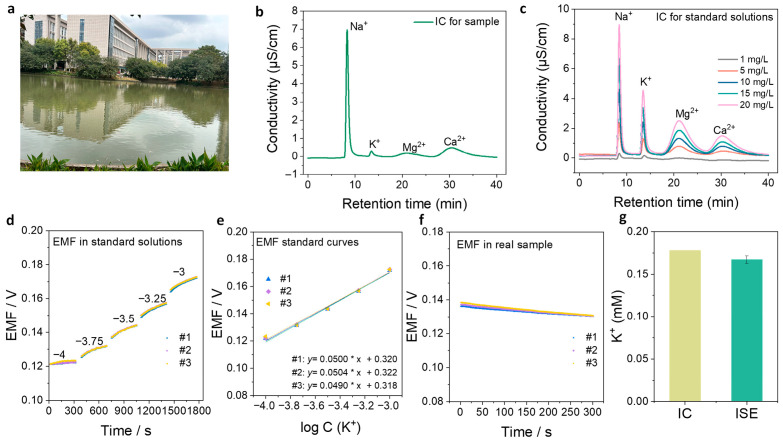
Practical sample analysis of K^+^: (**a**) The lake water sampling located near the university. (**b**) Ion chromatogram (IC) of the diluted lake water sample. (**c**) ICs of Na^+^, K^+^, Mg^2+^, and Ca^2+^ standard solutions. (**d**) EMF response curves of KMnFe(CN)_6_-based SC-ISEs toward K^+^ in the concentration range of 10^−4^–10^−3^ M in mixed background electrolytes containing Na^+^, Mg^2+^, and Ca^2+^. The numbers on the curves indicate the logarithmic concentrations of K^+^ (*n* = 3). (**e**) Corresponding EMF calibration curves with the average slope and intercept indicated in the figure (*n* = 3). (**f**) EMF response curves of KMnFe(CN)_6_-based SC-ISEs recorded in the real lake water sample (*n* = 3). (**g**) Comparison of K^+^ concentrations determined by ion chromatography (IC) and the SC-ISE method.

To evaluate the practical applicability of the KMnFe(CN)_6_-based SC-ISEs, K^+^ detection in a real lake water sample was carried out. The lake water was collected near the university campus and filtered prior to analysis (Figure 6a). Ion chromatography (IC) was first employed to characterize the ionic composition of the lake water sample, which revealed the presence of Na^+^, K^+^, Mg^2+^, and Ca^2+^ (Figure 6b). To determine the accurate concentrations of these ions, IC measurements were performed using a series of standard solutions (Figure 6c). Based on the corresponding calibration curves (Figure S12) and IC results for the lake sample (Table S3), the concentrations of Na^+^, K^+^, Mg^2+^, and Ca^2+^ in the lake water were determined to be 3.38 mM, 0.18 mM, 0.35 mM, and 1.05 mM, respectively. Subsequently, the KMnFe(CN)_6_-based SC-ISEs were employed to determine the K^+^ concentration in the lake water sample. The electrodes were first calibrated in the concentration range from 10^−4^ to 10^−3^ M with a step increment of 0.25 decades in a mixed Na^+^/Ca^2+^/Mg^2+^ background electrolyte to simulate the ionic environment of the lake water (Figure 6d). The resulting EMF calibration curve exhibited an average slope of 49.8 mV dec^−1^ and an intercept of 320 mV (Figure 6e). The electrode potential was then recorded in the actual lake water sample (Figure 6f). Based on the calibration curve, the K^+^ concentration in the lake water was calculated to be 0.17 ± 0.01 mM. Compared with the IC measurement result (0.18 mM), the relative error was approximately 5.6% (Figure 6g). This result demonstrates that the KMnFe(CN)_6_-based SC-ISEs can provide reliable and accurate K^+^ determination in real environmental water samples.

## 4. Conclusions

In summary, an ISM-free K^+^-SC-ISE was successfully developed using the KMnFe(CN)_6_ as the potentiometric sensing transducer material for K^+^ detection. Experimental results demonstrate that KMnFe(CN)_6_ exhibits a pronounced potentiometric response toward K^+^, confirming the feasibility of employing this single material as a bifunctional layer for simultaneous K^+^ recognition and ion-to-electron transduction. The KMnFe(CN)_6_-based electrode also shows favorable selectivity toward K^+^, enabling reliable detection even in complex environments such as real samples. Compared with conventional ISM-based SC-ISEs, the proposed ISM-free electrode eliminates the need for ionophore-containing membranes. In addition, the simplified two-layer architecture improves structural robustness while maintaining excellent sensing performance. Overall, this work highlights the potential of ISM-free SC-ISEs and provides an alternative strategy for the design of integrated ion-selective electrodes.

## Data Availability

The original contributions presented in this study are included in the article. Further inquiries can be directed to the corresponding author.

## Supplementary Materials

The following supporting information can be downloaded at https://www.mdpi.com/article/10.3390/membranes16050156/s1. Figure S1. FTIR spectrum of the as-prepared KMnFe(CN)_6_. Figure S2. TGA curve of the as-prepared KMnFe(CN)_6_. Table S1. ICP-MS element mapping and the determined chemical composition. Figure S3. Potential response curves toward interfering Li^+^. Figure S4. Potential response curves toward interfering Na^+^. Figure S5. Potential response curves toward interfering Ca^2+^. Figure S6. Potential response curves toward interfering Mg^2+^. Figure S7. Potential response curves toward interfering NH_4_^+^. Figure S8. pH effects for the KMnFe(CN)_6_ electrodes. Figure S9. Potential response curves toward interfering Mn^2+^. Figure S10. Electrochemical impedance spectra (EIS) for KMnFe(CN)_6_ electrodes. Figure S11. Water-layer test for the KMnFe(CN)_6_ electrode. Table S2. Comparison of analytical performances of potentiometric K^+^-ISEs. Table S3. Analysis results of ion concentrations in natural lake water samples by ion chromatography. Figure S12. Standard curves of Na^+^, K^+^, Mg^2+^ and Ca^2+^ ions were obtained by ion chromatography. Ref. [57] is cited in Supplementary Materials.
