# Supplementary material for: Potentiometric Solid-Contact K+ Ion-Selective Electrodes Based on the KMnFe(CN)6 Transducer
Source: Membranes (Basel). 2026 Apr 29;16(5):156. doi: 10.3390/membranes16050156 (PMC13208133; doi:10.3390/membranes16050156)
Supplement: Supplementary file 1 [file membranes-16-00156-s001.zip › membranes-4230603-supplementary.pdf]

Supporting Information

Potentiometric Solid-Contact K^+ Ion-Selective Electrodes Based on the $KMnFe(CN)_6$ Transducer

Huali Deng[1], Zhanhao Liu[1], LiNiu[1,2] and Shiyu Gan[1*]

[1]*Center for Advanced Analytical Science, Guangzhou Key Laboratory of Sensing Materials & Devices, Guangdong Engineering Technology Research Center for Photoelectric Sensing Materials & Devices, School of Chemistry and Chemical Engineering, Guangzhou University, Guangzhou 510006, P. R. China*

[2]*School of Chemical Engineering and Technology, Sun Yat-sen University, Zhuhai, 519082, China*

** E-mail address: ccsygan@gzhu.edu.cn*

Table of contents

Chemicals

Figure S1. FTIR spectrum of the as-prepared $KMnFe(CN)_6$.

Figure S2. TGA curve of the as-prepared $KMnFe(CN)_6$.

Table S1. ICP-MS element mapping and the determined chemical composition.

Figure S3. Potential response curves toward interfering Li^+.

Figure S4. Potential response curves toward interfering Na^+.

Figure S5. Potential response curves toward interfering Ca^{2+}.

Figure S6. Potential response curves toward interfering Mg^{2+}.

Figure S7. Potential response curves toward interfering NH_4^+.

Figure S8. pH effects for the $KMnFe(CN)_6$ electrodes.

Figure S9. Potential response curves toward interfering Mn^{2+}.

Figure S10. Electrochemical impedance spectra (EIS) for $KMnFe(CN)_6$ electrodes.

Figure S11. Water-layer test for the $KMnFe(CN)_6$ electrode.

Table S2. Comparison of analytical performances of potentiometric K^+-ISEs.

Table S3. Analysis results of ion concentrations in natural lake water samples by ion chromatography.

Figure S12. Standard curves of Na^+, K^+, Mg^{2+} and Ca^{2+} ions were obtained by ion chromatography.

Chemicals

The main used chemical are listed as follows: Potassium citrate (Aladdin, 98%), $MnSO_4·H_2O$ (Acros, 99%), $K_4Fe(CN)_6·3H_2O$ (Sigma-Aldrich, 99%), poly (vinylidene fluoride) (PVDF, Sigma-Aldrich), 1-Methyl-2-pyrrolidinone (NMP, Aladdin, 99%), KCl (Sigma-Aldrich, 99%), NaCl (Sigma-Aldrich, 99%), LiCl (Sigma-Aldrich, 99%), $CaCl_2$ (Sigma-Aldrich, 99%), $MgCl_2$ (Sigma-Aldrich, 99%) and NH_4Cl (≥99.999%, Aladdin). Other used chemicals are at least analytical grade. The distilled water (Watsons) was used for the preparation of aqueous solutions.

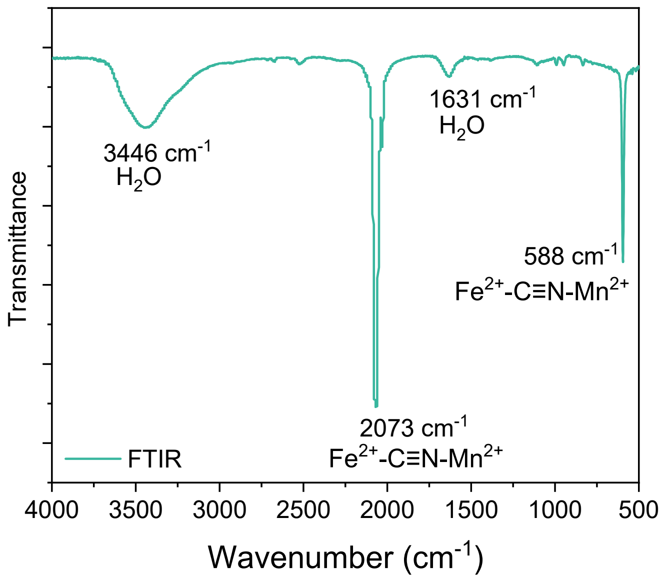

Figure S1. FTIR spectrum of the as-prepared $KMnFe(CN)_6$.

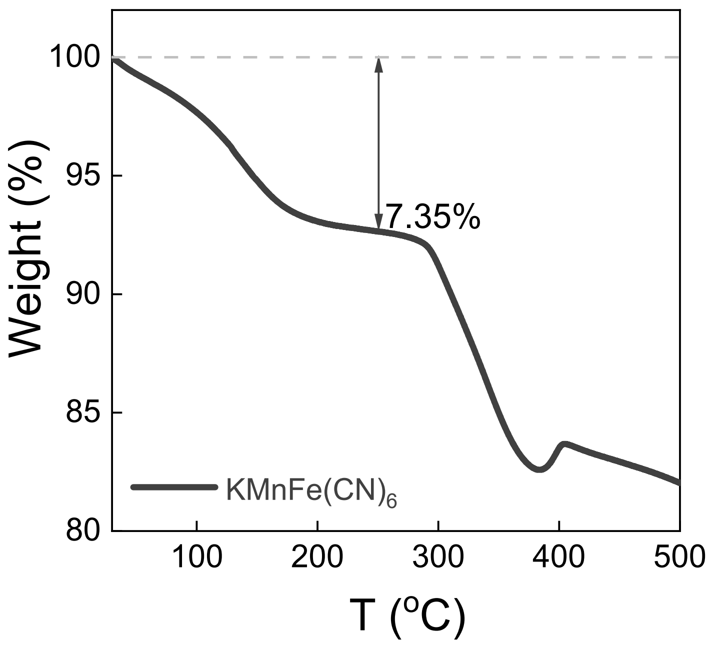

Figure S2. TGA curve of the as-prepared $KMnFe(CN)_6$.

Table S1. ICP-MS element mapping and the determined chemical composition.

Material	K (μg/L)	Mn (μg/L)	Fe (μg/L)	H_2O (wt%)	Chemical compositions
$KMnFe(CN)_6$	137	122	101	7.35%	$K_{1.58}Mn[Fe(CN)_6]_{0.81}$•$1.27H_2O$

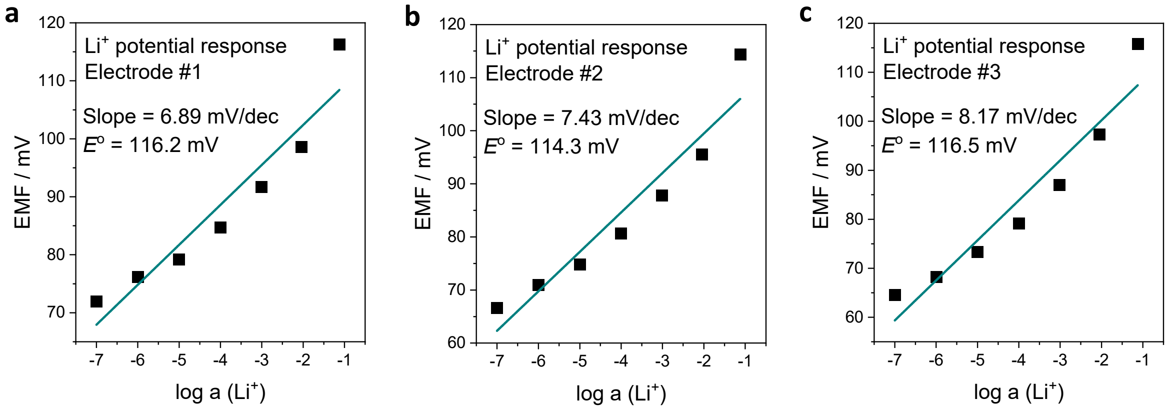

Figure S3. (a-c) Potential response curves toward interfering Li^+ by three electrodes (*n*=3).

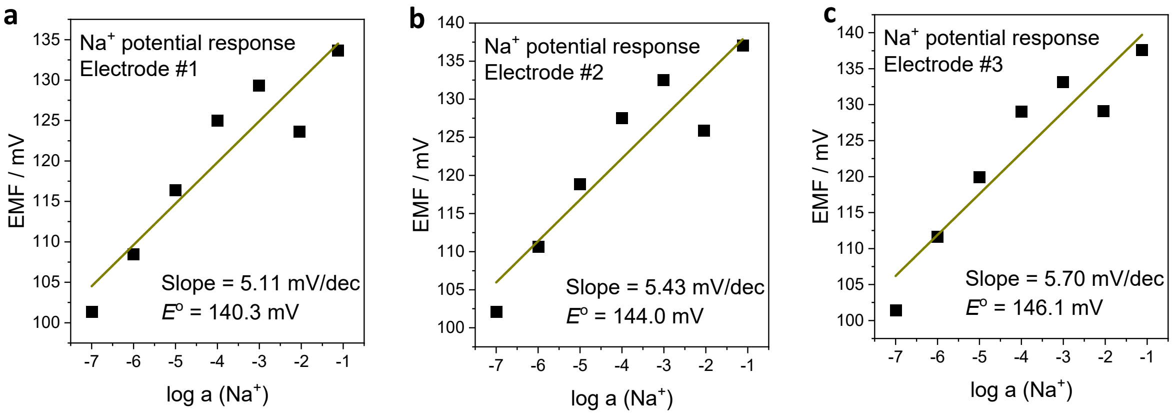

Figure S4. (a-c) Potential response curves toward interfering Na^+ by three electrodes (*n*=3).

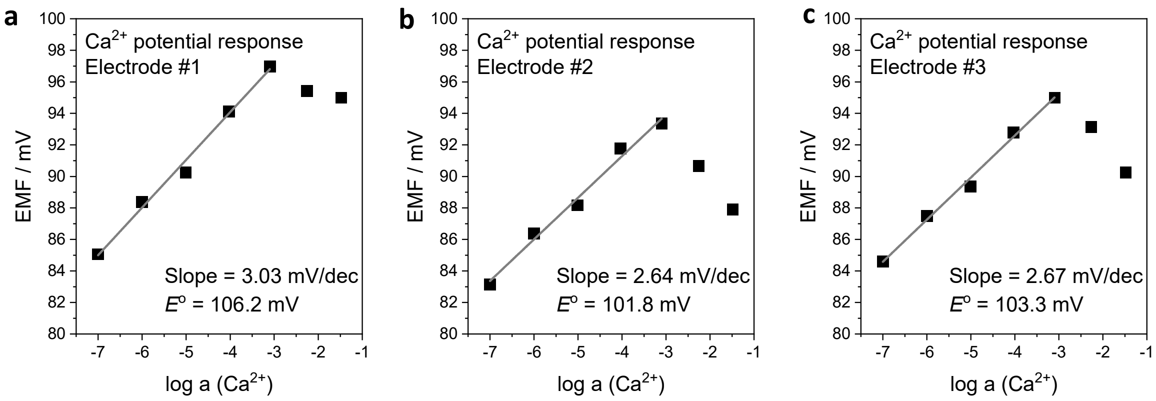

Figure S5. (a-c) Potential response curves toward interfering Ca^{2+} by three electrodes (*n*=3).

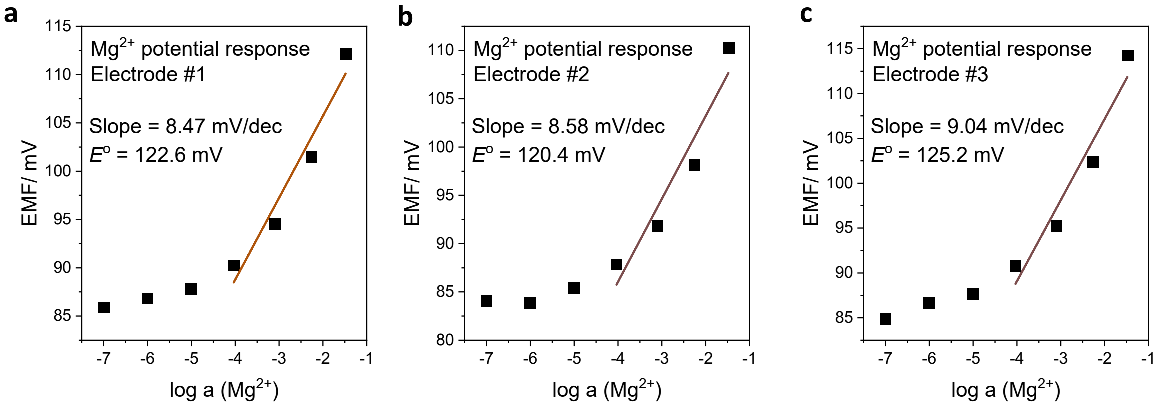

Figure S6. (a-c) Potential response curves toward interfering Mg^{2+} by three electrodes (*n*=3).

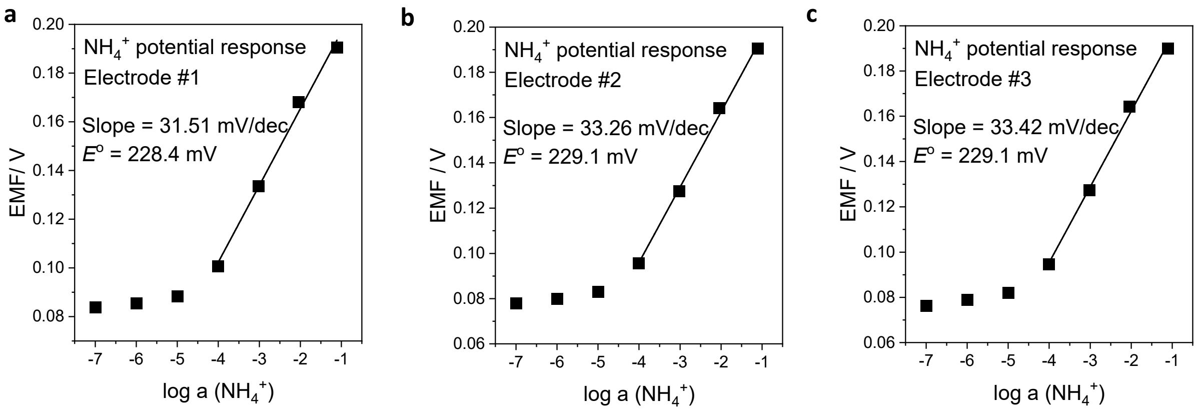

Figure S7. (a-c) Potential response curves toward interfering NH_4^+ by three electrodes (n=3).

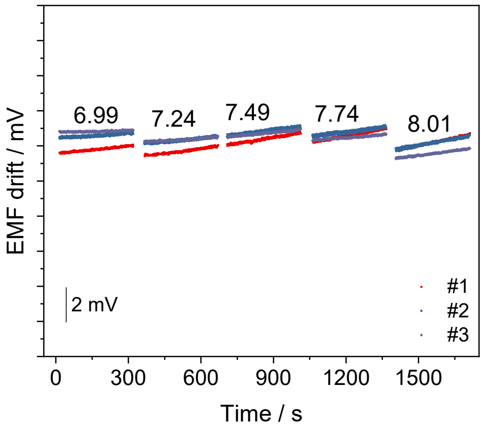

Figure S8. pH effects for the $KMnFe(CN)_6$ electrodes from pH 7 to 8 in 0.1 M KCl (n=3).

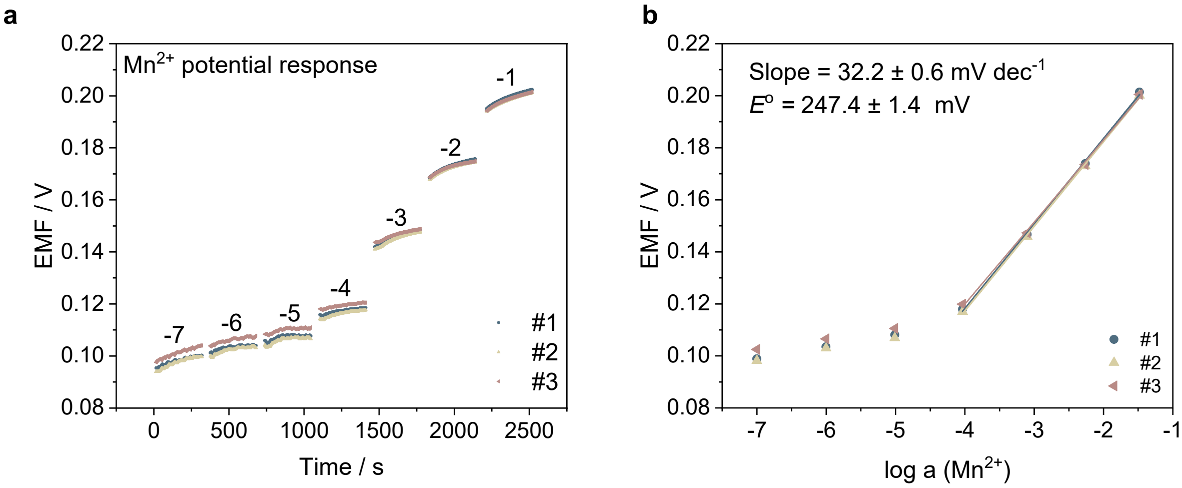

Figure S9. (a, b) Potential response curves toward interfering Mn^{2+} by three electrodes (n=3) and corresponding calibration curves.

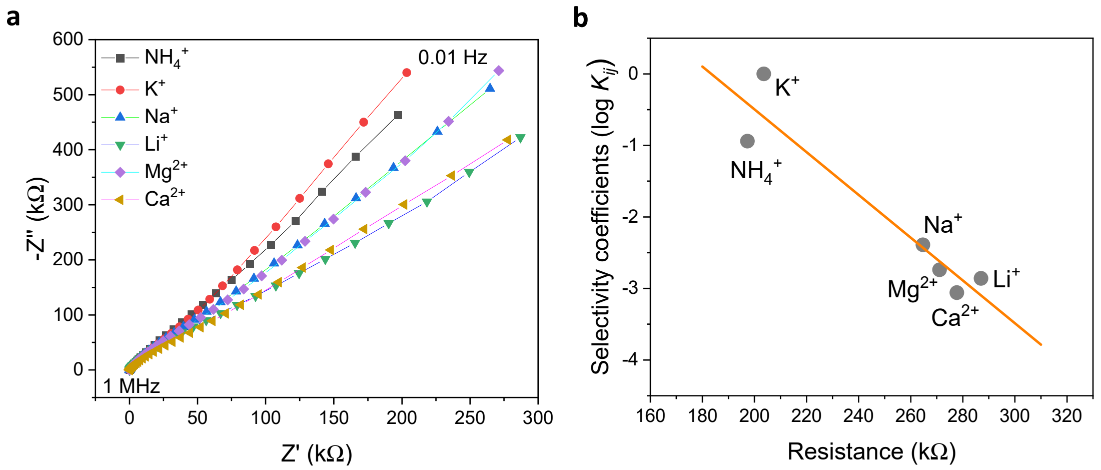

Figure S10. (a) Electrochemical impedance spectra (EIS) for $KMnFe(CN)_6$ electrodes in various electrolytes (0.1 M), NH_4Cl, KCl, NaCl, LiCl, $MgCl_2$, $CaCl_2$. EIS were carried out under open circuit potentials from 1 MHz to 0.01 Hz with the AC amplitude of 10 mV. (b) The correlation between selectivity coefficients and low-frequency resistance. The resistance is the real part of impedance at 0.01 Hz, which reflects the bulk membrane resistance and ion intercalation charge transfer resistance.

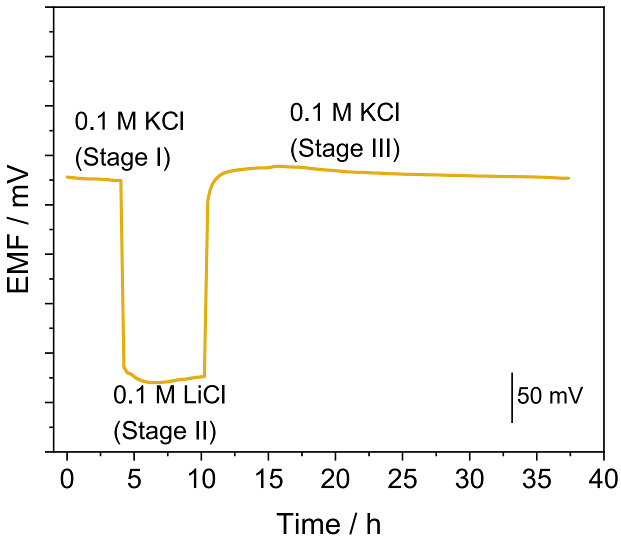

Figure S11. Water-layer test for the $KMnFe(CN)_6$ electrode. The EMF was first recorded in 0.1 M KCl (stage I), followed by 0.1 M LiCl (stage II) and returned 0.1 M KCl (stage III).

Table S2. Comparison of analytical performances of potentiometric K^+-ISEs.

K^+-ISEs	Sensitivity (mV/dec)	Selectivity (log K_{ij})	Linear range (M)	LOD (M)	Ref.
Valinomycin - based K^+-ISEs	49.52−54.00	Li^+ (−3.84), Na^+ (−3.51), Ca^{2+} (−3.96), Mg^{2+} (−3.84), NH_4^+ (−1.48)	10^{-4} to 10^{-1}	<10^{-4}	(1)
Valinomycin-based-K^+-ISFET	57.6	Li^+ (−4.1), Na^+ (−4.5), Ca^{2+} (−4.9), Mg^{2+} (−5.1), NH_4^+ (−2.4)	10^{-5} to 10^{-1}	10^{-7}	(2)
Nigericin-based K^+-ISEs	56	Li^+ (−2.2), Na^+ (−1.9), Ca^{2+} (−3.7), Mg^{2+} (−3.8)	$10^{-4.3}$ to $10^{-1.5}$	<$10^{-4.3}$	(3)
Bis(crown-ether)-based K^+-ISEs	43 ± 1	Li^+ (−5.0), Na^+ (−3.3), Ca^{2+} (−3. 0), Mg^{2+} (−2.6), NH_4^+ (−1.5)	10^{-4} to 1	7.9 × 10^{-5}	(4)
Crown-ether-based K^+-ISEs	/	Li^+ (−0.2), Na^+ (−1.4), Ca^{2+} (−1.80), Mg^{2+} (−1.40), NH_4^+ (−0.70)	/	/	(5)
Organo-phosphine based K^+-ISEs	/	Li^+ (−1.95), Na^+ (−2.35), Ca^{2+} (−3.05), Mg^{2+} (−2.90), NH_4^+ (−2.05)	10^{-5} to 10^{-1}	/	(6)
Calixarene-based K^+-ISEs	53-56	Li^+ (−3.7), Na^+ (−3.1), NH_4^+ (−0.75)	10^{-4} to 10^{-1}	/	(7)
$KMnFe(CN)_6$ without ISM	52.3 ± 1.0	Li^+ (−2.86), Na^+ (−2.39), Ca^{2+} (−3.06), Mg^{2+} (−2.74), NH_4^+ (−0.95)	10^{-4} to 10^{-1}	5.8 × 10^{-5}	This work

Table S3. Analysis results of ion concentrations in natural lake water samples by ion chromatography.

Ion	t_R (min)	Conc. (mg/L)	Peak Area [(μs/cm)*min]	Peak height (μs/cm)	Resolution	Tailing factor
Na^+	8.30	77.699	4.383	6.978	4.778	0.873
K^+	13.45	6.961	0.277	0.293	1.711	2.314
Mg^{2+}	20.97	8.351	0.829	0.196	1.285	1.215
Ca^{2+}	30.47	42.156	2.279	0.485	0.000	1.359

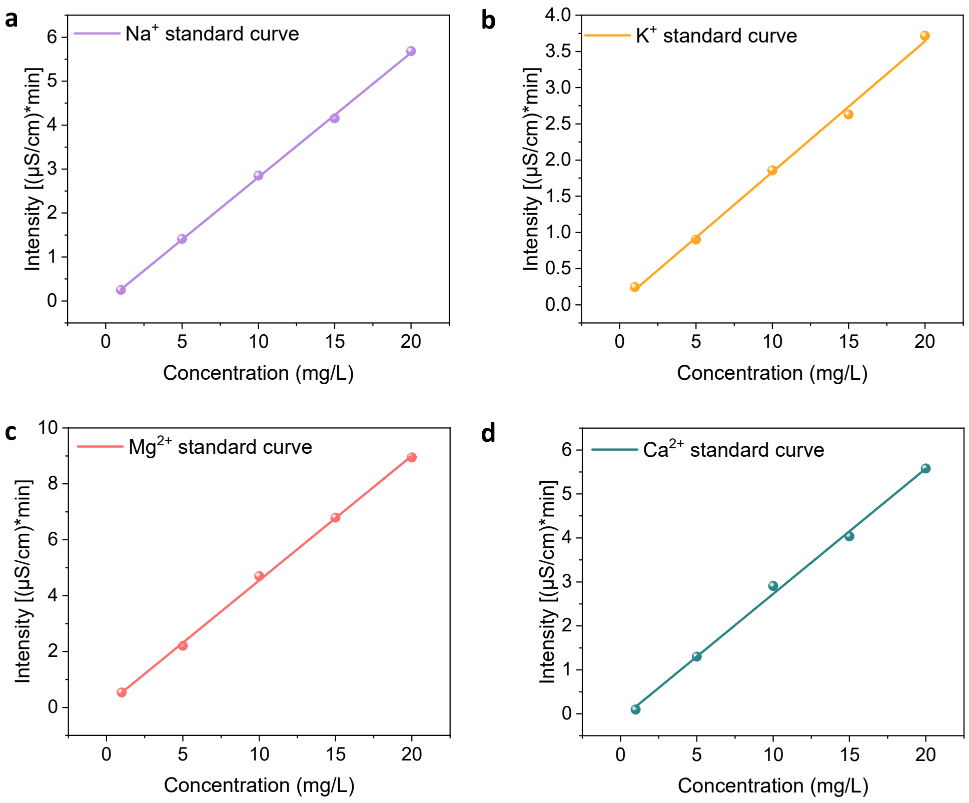

Figure S12. (a-d) Standard curves of Na^+, K^+, Mg^{2+} and Ca^{2+} ions were obtained by ion chromatography.

References

(1) Hong, S.; Kim, E.; Park, E.; Moon, S. M.; Chun, H. C.; Kim, Y.-R. Ion-selective electrodes for soil nutrient monitoring of potassium and nitrogen-related ions. *J. Electroanal. Chem.* **2025**, *999*, 119613.

(2) Högg, G.; Lutze, O.; Cammann, K. Novel membrane material for ion-selective field-effect transistors with extended lifetime and improved selectivity. *Anal. Chim. Acta* **1996**, *335* (1), 103-109.

(3) Suzuki, K.; Tohda, K.; Aruga, H.; Matsuzoe, M.; Inoue, H.; Shirai, T. Ion-selective electrodes based on natural carboxylic polyether antibiotics. *Anal. Chem.* **1988**, *60* (17), 1714-1721.

(4) An, H.; Wu, Y.; Zhang, Z.; Izatt, R. M.; Bradshaw, J. S. The synthesis of bis(benzo-crown ether)s and their incorporation into potassium-selective PVC membrane electrodes. *J. Inclusion Phenom. Mol. Recognit. Chem.* **1991**, *11* (4), 303-311.

(5) Wasilewski, J.; Biernat, J. F. Bis-substituted benzo-15-crown-5 ethers as ion carriers in potassium ion-selective electrodes. *J. Inclusion Phenom. Mol. Recognit. Chem.* **1991**, *10* (1), 109-118.

(6) Saleh, M. B.; Taha, F.; Aof, G. S. Potentiometric potassium selectivity of polymer membrane electrodes prepared with organo-phosphine ligands. *Electroanalysis* **1995**, *7* (8), 770-773.

(7) Sudhölter, E. J. R.; van der Wal, P. D.; Skowronska-Ptasinska, M.; van den Berg, A.; Reinhoudt, D. N.; Bergveld, P. Transduction of host-guest complexation into electronic signals: Favoured complexation of potassium ions by synthetic macrocyclic polyethers using membrane-modified, ion-sensitive field-effect transistors (ISFETs). *Recl. Trav. Chim. Pays-Bas* **1990**, *109* (3), 222-225.
